# The Demise of Poskanzer and Schwab's Influenza Theory on the Pathogenesis of Parkinson's Disease

**DOI:** 10.1155/2013/167843

**Published:** 2013-06-11

**Authors:** Danny Estupinan, Sunina Nathoo, Michael S. Okun

**Affiliations:** Department of Neurology, University of Florida Center for Movement Disorders and Neurorestoration, 3450 Hull Road, 4th Floor, Gainesville, FL 32607, USA

## Abstract

In 1961, David C. Poskanzer and Robert S. Schwab presented a paper, “Studies in the epidemiology of Parkinson's disease predicting its disappearance as a major clinical entity by 1980.” This paper introduced the hypothesis that Parkinson's disease was derived from a single aetiology, the influenza virus. We review the original Poskanzer and Schwab hypothesis that Parkinson's disease was based on the association between the 1918-19 influenza epidemic and the later observation of Parkinsonism in some influenza sufferers. We also further explore the prediction that Parkinson's disease would totally disappear as an entity once original influenza victims were all deceased. Current research has revealed that there are many potential causes and factors important in the occurrence of Parkinson's disease, postencephalitic Parkinsonism, and encephalitis lethargica. Poskanzer and Schwab presented a novel hypothesis; however, it was proven false by a combination of research and time.

## 1. Introduction

In 1817, James Parkinson, a London physician, wrote about six patients in *An Essay on the Shaking Palsy* and offered one of the first descriptions of Parkinson's disease. He described several symptoms of what he referred to as “paralysis agitans” and “involuntary tremulous motion… in parts not in action and even when supported; with a propensity to bend the trunk forwards… the senses and intellect being uninjured” [1]. During the mid-1800s, Jean-Martin Charcot also made several contributions to the early descriptions of Parkinson's disease including the addition of symptoms such as the masked facies, rigidity, bradykinesia, and postural as well as gait issues [2, 3]. In 1876, Charcot rejected the term “paralysis agitans” since patients were not physically weak and did not always exhibit tremor. He suggested naming the entity after James Parkinson [2]. He described Parkinson's disease as “névrose” or a neurological disease without a known pathologic lesion [2]. Charcot also separated the entity from other tremulous diseases such as multiple sclerosis [3]. 

In 1917, one hundred years after Parkinson's first description, the Romanian born Greek neurologist Constantin von Economo observed a cluster of unusual symptoms such as high fever, pharyngitis, confusion, lethargy, ophthalmoplegia, somnolence, and mental status changes. This confluence of symptoms was referred to as encephalitis lethargica (EL), a clinical phenomenon occurring in Europe and North America between 1916 and 1926, that had a wide range of associated signs and symptoms [4–7]. As many as twenty-eight types of EL were characterized by symptomatology by various physicians, including von Economo, who specifically described three distinct patterns, somnolent-ophthalmoplegic, hyperkinetic, and amyostatic-akinetic forms [8–10]. The latter form was associated with a chronic sequelae, a syndrome von Economo described ensuing months to years following resolution of initial symptoms [9, 10]. This syndrome included Parkinsonian features such as bradykinesia, masked facies, and resting tremor [4, 10]. Parkinsonism was also observed during the acute phase of the illness, but it was transitory in nature, suggesting the symptoms were evident in both acute and chronic phases of disease [9]. By 1927, 65,000 cases of EL had been reported, although the exact numbers remain unknown, and are difficult to ascertain due to the lack of official statistics and the polymorphic nature of disease [7, 9, 10]. The total number of EL cases ranged from 25,000–40,000 and 80,000–120,000 in the US and Western Europe, respectively [10]. One estimate suggested that EL was responsible for the death of over 500,000 people worldwide, suggesting a large mortality [11]. 

Patients who recovered from acute EL were reported to more likely develop postencephalitic Parkinsonism (PEP) as long-term neurological sequelae, usually 6 months to 1 year after an acute episode [10]. EL and PEP are, however, two distinct clinical entities, both of which can manifest with Parkinsonism [4, 7, 10, 12, 13]. EL, also referred to as “sleeping sickness,” and is considered an atypical form of acute encephalitis, affecting mainly the central nervous system with symptoms of headache, sore throat, chills, weakness, mild gastrointestinal disturbances, lethargy, sleepiness, and stupor [5–7, 10]. PEP manifests with symptoms such as rigidity, tremor, dysarthria, flexed postures, and masked facies [14]. PEP has been thought of by some experts to be a potential long-term complication of EL [10, 12]. 

In the 1920s and 30s, as PEP cases continued to emerge as a consequence of EL, the distinction between PEP and idiopathic Parkinson's disease blurred [10]. The symptoms of PEP overlap with idiopathic Parkinson's disease despite the clinical differences. Idiopathic Parkinson's disease and PEP differ in that PEP more commonly presents in middle age, usually with symptoms that last longer than a decade. PEP patients may manifest oculogyric crises, which are not often seen in idiopathic Parkinson's disease [12, 15]. In contrast, idiopathic Parkinson's disease more commonly presents in the 60's, and cases are not rapidly progressive [10]. Additionally, these two nosological entities can be considered distinct based on their respective neuropathology. Idiopathic Parkinson's disease is characterised by neurofibrillary tangles, Lewy bodies and other inclusions, and also neuronal loss in the substantia nigra and locus coeruleus [10, 16]. PEP, in contrast, shows widespread neurofibrillary degeneration and gliosis of the substantia nigra without Lewy bodies [10, 16]. EL neuropathology reveals acute inflammation in the grey matter, superficial congestion, widespread neurofibrillary tangles, and lymphocytic infiltration into the basal ganglia [10, 17]. 

PEP cases increased during the 1920s during the aftermath of the EL epidemic, leading to the widespread idea that “all cases of Parkinsonism were ultimately caused by EL” [10]. The etiology of EL, although agreed on to be likely infectious, has continued to be a subject of contention. Historically, the virus from the 1918 Spanish influenza pandemic became widely accepted as the likely cause of EL simply due to a temporal association [10, 12, 17, 18]. Recent direct evidence, however, refutes this initial hypothesis that EL was due to influenza virus infection. The precise cause of EL remains unknown [10]. EL has not recurred in the subsequent influenza pandemics but continues to be an interesting clinical entity, especially with respect to its relationship to Parkinsonism. EL, PEP, and idiopathic Parkinson's disease are thought to represent distinct clinical entities, but their respective roles in the possible pathogenesis of Parkinsonism are still hotly debated [18]. The confusion between influenza, EL, and PEP and a potential association with Parkinson's disease has fuelled much of the speculation about influenza and the development of future Parkinson's disease.

In the 1950s and 60s, two Harvard neurology based faculty, David C. Poskanzer and Robert S. Schwab, put forth a bold hypothesis that Parkinson's disease was due to influenza and that Parkinson's disease as an entity would “die out” by 1980. The Poskanzer and Schwab hypothesis (PSH) linking influenza infection with Parkinsonism after the 1918 influenza pandemic was put forth in 1956 [19]. According to Poskanzer, one of the original inspirations for his hypothesis was derived from this 1956 study that reported Parkinson's disease prevalence shifting towards an older age group when compared to a previous seven-year period. Sixty percent of these patients recollected a history of influenza infection and there was only a single patient born after 1927 [19]. Poskanzer was struck by the possibility that a viral infection could possibly be the underlying cause of Parkinson's disease [19, 20]. In 1961 at the 86th American Neurological Association (ANA) meeting held in Atlantic City, New Jersey, Poskanzer and Schwab presented a paper, “Studies in the epidemiology of Parkinson's disease predicting its disappearance as a major clinical entity by 1980”[21]. This paper formally presented the hypothesis that Parkinson's disease was caused by previous influenza viral infection. The paper and the hypothesis were both received with scepticism [15]. The paper received public attention as early as 1962 in a New York Times article that linked “palsy to virus” as researchers believe Parkinsonism would die out in 20–40 years [22]. Later in 1963, Poskanzer and Schwab published studies postulating a direct link between viral exposure and Parkinson's disease [23]. The two neurologists reported that the cohorts of Parkinson's disease patients exposed to influenza during two successive pandemics (1920–24 and 1955–59) had the greatest Parkinson's disease incidence. As a result of these collective observations, the two neurologists put forth a bold assertion that the incidence of Parkinson's disease would dramatically tail off and perhaps even disappear with the death of all influenza sufferers [23]. Poskanzer was so confident of his theory that in a 1974 TIME magazine article entitled The Parkinson's Puzzle, he famously challenged “I offer a bottle of scotch to any doctor in the U.S. who can send me a report of a clearly diagnosed case of Parkinson's in a patient born since 1931. So far it's cost me 14 bottles—just 14 of these younger patients identified since 1961” [24]. Although the modern understanding of EL, PEP, and influenza remains debated, the number of Parkinson's disease cases has continued to grow despite “the dying off” of the original influenza pandemic victims. We aim in this paper to review the Poskanzer and Schwab hypothesis (PSH), its historical context, and the current overall prevalence of Parkinson's disease post-1980.

## 2. Methods

A complete PubMed review of the literature on EL, influenza, and PEP (search terms encephalitis lethargica and influenza, encephalitis lethargica and post-encephalitic Parkinsonism, influenza and post-encephalitic Parkinsonism) revealed case reports, research papers, and literature reviews. An examination of the available literature including citations from these papers revealed multiple descriptions of EL and PEP. Additionally, papers which commented on the relationship between influenza and PEP were also reviewed [4, 7, 11–13, 25, 26]. Also examined were the Proceedings of the ANA, the 1963 Poskanzer and Schwab paper, an “Analysis of Parkinson's syndrome: Evidence for a single aetiology related to Subclinical Infection about 1920,” and the 1956 Schwab paper, “Shift to older age distribution in Parkinsonism; a report on 1,000 patients covering the past decade from three centers” [12, 19, 23]. A PubMed search was also performed for papers that cited the 1963 paper. We reviewed the epidemiological studies that sought to verify the PSH. Finally, we reviewed current and projected statistics provided by Dorsey's recent work, as well as other major epidemiological papers on Parkinson's disease [5, 6, 14, 18, 27–29].

## 3. Results

The original cohort analysis was performed using all cases of Parkinson's disease (*n* = 1383) seen at Massachusetts General Hospital from 1875 to 1961 [23]. The Poskanzer and Schwab prediction in the 1963 paper was that “as the cohort affected by Parkinson's syndrome aged and died off, the number of cases of Parkinson's disease should diminish markedly” [23]. The cohort he was referring to included individual cases of Parkinson's syndrome exposed to the influenza virus during the 1918 pandemic [19, 21]. However, approximately 11.2% of this cohort was known to present with encephalitis during the periods 1920–24 and 1955–59. Between these periods of time, or 35 years, the mean age at the onset of Parkinson's disease increased by 27 years [23]. Additionally, his cohort of patients was all drawn from ages 5–59 and many patients presented with Parkinsonism of unknown cause, although likely postencephalitic in origin, in congruence with the cohort hypothesis itself [23]. The 1956 data also suggested a common infection despite only 10% of their population giving a history of EL, and 51% giving a history of association with the 1918 influenza virus [19, 23]. The neurologists postulated an insult affecting their cohort no later than 1920. Despite the lack of history of encephalitis, they suggested a possible subclinical infection that went undiagnosed [19, 23, 30]. Their hypothesis held that by 1980 the incidence of Parkinson's disease would greatly diminish as individuals belonging to the influenza pandemic died. Their projection of a precipitous drop in cases was “presented only as an interesting exercise with full knowledge of its potential inaccuracy” [23]. Subsequent investigators published epidemiological reports that supported their hypothesis [31, 32]. Brown and Knox, Kaplan, and Leibowitz and Feldman all published data that upheld the cohort hypothesis [31–35]. However, Kessler, Nobrega, Hull, and Kurland later disagreed as there was little change in the mean age at onset [30, 32, 36–39]. A study in 1976 in Warsaw (*n* = 495) examined patients treated for Parkinsonism in 1972–6 and also failed to confirm the hypothesis [40]. Epidemiological data suggest that cases of Parkinson's disease increased rather than decreased during this period [26, 27, 41]. 

The current projection is that Parkinson's disease prevalence will continue to increase. Dorsey et al. recently commented that, based on the published prevalence studies, the number of individuals with Parkinson's disease in the 8 most populated nations (over age 50) was currently between 4.1 and 4.6 million in 2005. Dorsey further predicted that the number would roughly double from 8.7 to 9.3 million by 2030 [27]. 

## 4. Discussion

### 4.1. Poskanzer and Schwab Hypothesis: Support and Reception

The Poskanzer and Schwab prediction about the decline in Parkinson's disease incidence was false. The mean age of onset of Parkinson's disease increased since the influenza pandemic. Marttila and Rinne, Schrag and Schott, and Kurland argued that the increase was due to an aging population [31, 41, 42]. There may have also been a bias in the original influenza data that “reflected changes in medical practice and particularly specialty services, which have been sought by and become available to the elderly patient” [41]. The mean age of onset was 50.8 years, consistent with other studies [15]. However, no subset analysis was performed for patients with a history of encephalitis in order to determine age of onset, although cases with onset between 1915 and 1919 were examined individually largely due to the association with EL and pandemic influenza. This analysis revealed a mean age of onset at 34 years of age and was consistent with a study that performed a subanalysis on patients with secondary Parkinsonism associated with EL [15]. 

Two entities have been recognized since the pandemic: (1) Parkinsonism secondary to EL or postencephalitic and (2) idiopathic Parkinson's disease unrelated to influenza infection [30, 42, 43]. Younger patients with Parkinsonism secondary to EL were hypothesized to continue to die out while idiopathic Parkinson's disease cases would increase [44]. The PSH supported the idea that all PD was due to viral infection and that there was a subclinical infection that could contribute to symptom onset 40 years after the initial infection [23]. Even in cases of idiopathic PD, EL may have possibly been the underlying cause [11, 23]. Poskanzer and Schwab themselves stated that “it is almost impossible to differentiate EL from the influenza epidemic,” suggesting the inability to discern EL or influenza as the viral aetiology of their proposed subclinical infection [23]. They commented on the neuropathological changes in EL particularly those occurring in the substantia nigra and basal ganglia, both areas known to be affected by Parkinson's disease [23]. Gamboa et al. observed an association between a birth cohort in women in their late teens and 20s and the later development of Parkinsonism [45]. These patients were at an age most at risk for developing EL and had a greater risk of developing Parkinsonism, and this cohort supported the PSH [45]. 

The PSH hypothesis was initially dismissed by neurologists, who felt that PEP and idiopathic Parkinson's disease were separate entities [46]. Furthermore, the epidemiological studies mentioned previously failed to reveal the same cohort effect as Poskanzer and Schwab. Hoehn found that the modal age of onset from 1946 to 1976 was less than a decade higher than in the late 1890s and early 1900s, suggesting the same disease affected parkinsonian patients in old and modern eras, making the PSH less likely [43]. Recent analysis of data from England and Wales failed to reveal a birth cohort effect. Persons born around 1900 were more likely to die from Parkinson's disease than people born before 1888 or after 1924 [46]. Another study based on Swiss mortality data suggested that birth cohort effects on Parkinson's disease were greater for people born before 1920 [47]. Examination of mortality data also revealed a higher mortality from Parkinson's disease in people born around the late 1880s and early 1900s. The maximum death rate in the cohort occurred between 1906 and 1910 [48]. These findings supported the idea of a causative agent underlying Parkinson's disease; an agent that acted acutely and disappeared rapidly and could have possibly been EL [46]. Mortality data for Parkinson's disease showed a reduction in the 1970s with the introduction of levodopa [44]. Despite being false, the PSH was a milestone in that it was the formulation of a theory regarding Parkinson's disease etiology [49].

The PSH itself was put forth boldly by two neurologists who were pioneers during their time. Schwab was “by chance” involved in several important developments including administration of neostigmine for myasthenia gravis, founding the first clinical laboratory for recording electroencephalograms, and pioneering the use of levoamphetamine for narcolepsy. Schwab also was the first to use apomorphine for Parkinson's disease treatment prior to L-dopa therapy [50]. A former student described Poskanzer as imaginative with challenging, creative ideas in neurology inclusive of his theory of mild or subclinical infection as a cause of Parkinsonism [20]. Poskanzer and Schwab together initiated the treatment of Parkinson's disease with amantadine after observing a 58-year-old patient who had symptomatic improvement with the drug which was prescribed to prevent influenza [50, 51]. 

### 4.2. Encephalitis Lethargica and Influenza

The PSH was based on the idea that there was a subclinical infection prior to 1920, with EL, a potential cause identified by the authors and perhaps a cause that was more likely than influenza [23]. Therefore, discussion of EL such as its historical context, potential aetiologies, and implications is warranted. Contemporary observers of the EL epidemic maintained that both the EL and the influenza epidemics were not connected, despite the popularity at the time of the idea that the influenza virus was the cause of EL [10]. The medical profession at that time simply viewed EL as a form of influenza [18]. The current prevailing viewpoint is that EL and the 1918 influenza pandemic were not related etiologically [10, 11]. von Economo ultimately concluded that EL was a separate disorder. EL preceded the 1918 influenza pandemic, had a distinct clinical picture and unique pathology. EL was associated with midbrain lesions, while influenza was associated with pulmonary lesions [8, 10, 18, 52]. 

Additionally, there was epidemiological evidence that suggested that the 1918 influenza virus originated in USA, and was transported to Europe by American troops in World War I [53]. EL actually spread in the opposite direction from Europe to North America. It is however possible that there was an EL-like syndrome that went unrecognized during the time since public interest was centred on World War I [11]. Interestingly, the years of higher occurrence of EL coincided with a drop in influenza cases, suggesting a weak correlation [53]. 

Timelines revealed inconsistencies, as influenza spread in weeks, and EL over months [10]. Historically, EL-like disorders have been reported during previous influenza epidemics, such as the *nona* pandemic in Italy in the 1890s [7, 17, 54, 55]. On the basis of 1889 influenza being associated with certain nervous manifestations, some authors assumed all nervous symptoms were attributed to influenza [17]. However, no syndrome resembling EL occurred in the two influenza pandemics after 1918 (e.g., 1957 and 1968) suggesting that a unique type of virus was required to produce the array of neurological symptoms associated with EL [10]. Finally, there was a lack of influenza history in two thirds of EL patients, supporting the notion the two were not related [11].

Due to the temporal association between EL and influenza, it was assumed that influenza must be the cause. It is however possible that EL was actually due to another virus, or due to an infective agent that was concurrently circulating with influenza. If influenza was not the cause of EL, then what was? The aetiology of EL remains a mystery, although there are several theories concerning possible causes. Such theories include viruses—either a neurotropic virus different from influenza (e.g., polio), or activation of a latent virus, bacteria—poststreptococcal-like illness analogous to chorea and rheumatic fever, toxins, dietary issues due to wartime deprivation, miscellaneous, or “rag bag” diagnosis with the actual incidence being inflated by other conditions, or an autoimmune reaction to a virus [7, 10, 28, 56, 57]. 

The relationship between EL and influenza has been examined historically and scientifically, with most EL researchers maintaining that influenza is an unlikely cause of EL [10, 11, 52]. Others suggest that the association cannot be ruled out [7, 12, 58]. No gold standard titer testing was available at the time, making diagnosis of EL and influenza subjective and based on clinical findings alone [12, 58]. The supervisor of the vaccine trials for EL and a major contributor to the Matheson commissioned EL literature survey Josephine Neal in 1942 commented “the range of symptomatology in acute EL was so wide that often the diagnosis could be made only with difficulty and occasionally not with certainty” [12]. There were many limitations in the available cases in the literature. Ultimately, most diagnoses in the literature of both EL and PEP were post hoc, recounted a plethora of symptoms, and attributed the symptoms to various aetiologies [4, 12–15, 19]. Many reported cases of influenza were likely biased by patient recall. The 1918 influenza epidemic frequently resulted in cases where reports of neurological symptoms were identical to EL (i.e., diplopia, ptosis, paralyses, or psychoses), making EL a subjective diagnosis that was difficult to separate from influenza alone [12]. Lethargy could result from either EL or influenza [12, 17]. All of the reports of EL and of influenza were retrospective and unblinded, demonstrating the difficulty of ascertaining which cases were which [12, 13, 25]. 

Given the lack of advances in virology during the pandemic, objective diagnosis of influenza was not possible. There is a lack of direct evidence from serological, PCR, or antibodies that link influenza and EL, with all studies limited by the amount of EL material available [11]. Studies that have used PEP tissue, which is more readily available than EL tissue, have not confirmed influenza as more likely occurring in idiopathic Parkinson's disease [11]. Study of these dated specimens is troublesome due to the lack of temperature control and autolysis due to lack of postmortem refrigeration [55]. Decades later, archived EL brain specimens when carefully examined had not revealed evidence of influenza RNA [10, 11, 25, 59]. Attempts to reproduce EL from postmortem brain extracts have been failures [7]. One study demonstrated direct antibody immunofluorescence for the neurotropic influenza virus A antigen within in the hypothalamus in six human PEP brains. In the same study, there was, however, no antibody reaction in five postmortem human cases of idiopathic Parkinson's disease [45]. A human postmortem study of substantia nigra depigmentation in young victims of EL suggested that an infectious aetiology may have been responsible for the Parkinsonism symptoms [60]. Basal ganglia autoimmune reactions have been shown in 90% of cohort of 20 postmortem patients known to have suffered from EL [28]. These patients had bradykinesia, rigidity, or resting tremor suggesting the parkinsonian phenotype and an autoimmune mechanism [28]. 

### 4.3. Postencephalitic Parkinsonism

Poskanzer and Schwab considered PEP as a major potential etiology of Parkinsonism, although only 11.2% of their cohort reported a history of encephalitis. Cases of PEP increased during the 1920s and 1930s, and this helped to propagate the idea that all cases of Parkinsonism were caused by EL [10]. Studies suggested that 50% of PEP patients had acute EL [10]. One study concluded that 15% of PEP cases did not have a history of acute EL, and only few cases had asymptomatic EL [12]. Development of PEP was not only attributable to EL, but may have also been due to a complex interplay of environmental and genetic factors [12]. In the medical practices of 1930s and 1940s, typical cases of idiopathic Parkinson's disease may have been defined as PEP with only a history of influenza identified by the doctor or identified as PEP due to a younger age at onset, blurring the lines between PEP and idiopathic disease [10, 61]. 

### 4.4. Viral Infection and Parkinson's Disease

The PSH shed light on the possibility of viral illness as a cause for Parkinsonism. Viral infection has today been shown to be a possible aetiological agent associated with the later development of Parkinson's disease. A number of viruses have been implicated and are not limited to influenza, coxsackie, herpes, western equine encephalitis, Japanese encephalitis B, and HIV [8, 18, 60]. One study revealed a clustering of patients who developed idiopathic Parkinson's disease if born during the influenza pandemic periods of 1890–1930. It was also suggested, but never proven, that a link existed between intrauterine influenza infection and Parkinson's disease [62]. Though it has been rare for researchers to recover evidence of influenza in brain tissues, it is possible that influenza resulted in damage to developing substantia nigra and this could have provided the nidus or first hit rendering a patient susceptible to the later development of Parkinson's disease [62]. One study has claimed that the risk of developing Parkinson's disease increases with the number of influenza attacks, though this has also never been confirmed [63]. A study in mice did however reveal an accumulation of viral antigen in the substantia nigra after intracerebral inoculation, suggesting that influenza A could cause Parkinsonism [64]. However, the more reasonable explanation is that exposure to viruses may be a first “hit” in a two hit hypothesis that could predispose one to develop Parkinson's disease due to sensitization [8]. Serological studies have failed to show any differences between idiopathic Parkinson's disease and controls in antibody titres, suggesting that age at infection, rather than infection itself may be important [65].

## 5. Conclusion

The PSH that Parkinson's disease would diminish or disappear as a particular cohort died was false. The original hypothesis that Parkinson's disease was due to subclinical infection due to an exposure prior to 1920 was compelling given the increase in Parkinsonism seen during the 1920s and 1930s. The birth cohort had a mean age similar to that of patients affected with EL, suggesting that these patients were exposed to a similar agent [23]. There were many reasons why during the first half of the twentieth century there was an idea that Parkinsonism could be due to a viral etiology. EL and PEP were assumed to be influenza or influenza related historically, but these relationships were never proven [12–14, 18, 19, 55, 59, 62, 63]. Today, most people who develop Parkinson's disease have had no one specific cause identified. Influenza may, however, provide the first “hit” that may lead to the later development of Parkinson's disease, suggesting a possible mechanism for viral infection in disease manifestation. More importantly, despite discounting Poskanzer and Schwab's initial hypothesis, the association between virus exposure and Parkinson's disease is still being actively pursued. Parkinson's disease has now outlived Poskanzer and Schwab's postinfluenza eradication theory; therefore new hypotheses to elucidate potential causes are warranted to explain why the incidence has increased, rather than decreased, as previously suggested.
